# Prevalence of Ivermectin use to prevent COVID-19 during the pandemic in Mato Grosso: cross-sectional home-based study

**DOI:** 10.1590/1980-549720230026

**Published:** 2023-05-08

**Authors:** Nathalia Beatriz Lobo da Silva, Roseany Patrícia da Silva Rocha, Amanda Cristina de Souza Andrade, Ana Cláudia Pereira Terças-Trettel, Ana Paula Muraro

**Affiliations:** I Universidade Federal de Mato Grosso, Institute for Collective Health – Cuiabá (MT), Brazil.; II Universidade do Estado de Mato Grosso – Tangará da Serra (MT), Brazil.; III Universidade Federal de Mato Grosso, Graduate Program in Collective Health – Cuiabá (MT), Brazil.

**Keywords:** COVID-19, Off-label use, Ivermectin, Coronavirus, Cross-sectional study, COVID-19, Uso off-label, Ivermectina, Coronavírus, Estudos transversais

## Abstract

**Objective:**

To analyze the use of ivermectin as COVID-19 prevention method by the population of Mato Grosso in 2020.

**Methods:**

This is a home-based survey, carried out between September and October 2020, in 10 pole cities of the socioeconomic regions of State. The use of ivermectin was evaluated through the question: “Did you take ivermectin to prevent COVID-19?”. Sociodemographic variables (sex, age group, education, family income), current work situation, being benefitted by government financial programs, as well as symptoms, seroprevalence of antibodies against SARS-CoV-2, and previous diagnosis of COVID-19 were evaluated. Prevalence and their associations were estimated using the chi-square test.

**Results:**

4.206 individuals were evaluated for prevalence of ivermectin use; 58.3% of the individuals responded positively, this rate being higher in the municipalities of the western region of the state (66.6%). There was no significant difference between sexes, but the prevalence was higher among people aged 50–59 years (69.7%), who were white (66.5%), with complete higher education or more (68.8%) and higher family income (≥3 minimum wages-64.2%). The use of this drug was even higher among participants who considered their knowledge of the disease good or very good (65.0%), who reported having symptoms of COVID-19 (75.3%), and who had been previously diagnosed with the disease (91.2%).

**Conclusion:**

There was a high prevalence of use of ivermectin as a method to prevent covid-19 by the population of Mato Grosso, indicating the need for strategies to inform the population about the risks of off-label use of drugs and to combat the advertising of drugs that are ineffective against COVID-19.

## RESUMO

**Objetivo:**

Analisar a utilização de ivermectina como prevenção da COVID-19 pela população de Mato Grosso em 2020.

**Métodos:**

Trata-se de um inquérito de base domiciliar, realizado entre setembro e outubro de 2020, em dez municípios-polos das regiões socioeconômicas do estado. O uso da ivermectina foi avaliado por meio da pergunta: “Tomou ivermectina para prevenir a COVID-19?”. Foram avaliadas as variáveis sociodemográficas (sexo, faixa etária, escolaridade, renda familiar), a situação de trabalho atual, o recebimento de benefícios financeiros governamentais, bem como sintomas, a soroprevalência de anticorpos contra SARS-CoV-2 e o diagnóstico prévio de COVID-19. As estimativas de prevalência e suas associações foram realizadas por meio do teste χ^2^.

**Resultados:**

Foram analisados 4.206 indivíduos, e a prevalência de uso de ivermectina foi de 58,3%, sendo maior nos municípios da região Oeste (66,6%). Não houve diferença significativa entre os sexos, a prevalência foi maior na da faixa etária de 50–59 anos (69,7%), em pessoas brancas (66,5%), com ensino superior completo ou mais (68,8%) e maior renda familiar (≥3 salários-mínimos — 64,2%). A utilização do medicamento ainda foi maior entre os que consideraram seu conhecimento sobre a doença como bom ou muito bom (65,0%), entre os que referiram ter apresentado sintomas de COVID-19 (75,3%) e que foram diagnosticados com a doença previamente (91,2%).

**Conclusão:**

Verifica-se a elevada a prevalência do uso de ivermectina para a prevenção da COVID-19 pela população de Mato Grosso, indicando a necessidade de estratégias para informar a população sobre os riscos do uso off-label de medicamentos e combater a publicidade de medicamentos sem eficácia contra COVID-19.

## INTRODUCTION

In January 2020, the World Health Organization (WHO) declared the outbreak caused by the SARS-CoV-2 virus as a public health emergency of international concern, and on March 11, 2020, COVID-19 was characterized by the WHO as a pandemic. In this context, a phenomenon called infodemic was also configured, which describes the increase in flow of information about a subject—some accurate, others not—, which makes it difficult for the population to select reliable information^1^.

When in excess, information can be used not to help the population, but to manipulate it according to interests of third parties^2^. In the first year of the COVID-19 pandemic, when there were still no approved vaccines for its prevention and treatments were being evaluated, a widely publicized subject was the use of drugs without scientific evidence^3^, such as hydroxychloroquine or chloroquine, azithromycin, ivermectin, and nitazoxanide, in addition to zinc and vitamins C and D supplements^4^.

The Ivermectin, despite not having proven efficacy against the new coronavirus^5^, was specifically recommended by doctors, health departments, and the federal government as part of a so-called early treatment, which led this drug to a sales peak that moved the revenue of their laboratories. Sales of ivermectin jumped from BRL 44.4 million in 2019 to BRL 409 million in 2020, an increase of 829%^4,6^. In Mato Grosso, for example, a so-called “COVID kit” was adopted, which included drugs without proven efficacy for COVID-19^7^. In the state capital, for example, the use of the kit was regulated in health units, and patients were instructed to read and sign a Term of Free and Informed Consent to receive drugs as a treatment without proof of effectiveness against COVID-19^8^.

Searches in the literature show few studies that have evaluated the profile of people who used ivermectin as a means of prevention against COVID-19. Only the population-based study in Manaus estimated this use, noting that 38% of people self-medicated to prevent or treat COVID-19, and 31% of them used ivermectin. The proportion of self-medication was even higher among those who had a previous diagnosis of COVID-19 (73%), and 67.5% of them used ivermectin^9^. Knowing the profile of users of this drug can contribute to understanding the phenomenon that occurred in the first year of the COVID-19 pandemic with off-label use of drugs without proven efficacy. The present study aimed to analyze the use of ivermectin as a method for COVID-19 prevention by the population of Mato Grosso in 2020, the first year of the pandemic.

## METHODS

This is a home-based survey carried out between September and October 2020 in ten hub municipalities in the socioeconomic regions of the state, the main cities being: Cuiabá, Várzea Grande, Cáceres, Rondonópolis, Barra do Garças, Tangará da Serra, Alta Floresta, Água Boa, Juína, and Sinop. A cross-sectional design was adopted, with cluster sampling in three stages: census sector (selected with probability proportional to the number of permanent households, according to data from the 2010 census); households (selected based on systematic sampling); residents over 18 years old (one randomly selected resident)^10^.

The sample was estimated at 4,530 individuals, considering the 2019 population estimate in selected municipalities for sample calculation (sample space: 1,650,643 inhabitants of the ten municipalities participating in the study), adopting a confidence level of 95% (95%CI), effect design of 1.5, 3% prevalence of seroprevalence of antigens against SARS-CoV-2 and precision of 0.65%. The sample size was calculated using the OpenEpi tool^11^. The percentage of 13% recomposition was added to the sample size, considering anticipated losses from refusals and closed households during visits. More details about the study design can be seen in Oliviera et al.^10^.

Data was collected by professionals from the municipal and state health departments, as well as students from courses in the health field at partner universities, after attending training to standardize the interviews. During data collection, the selected census tract was covered following a system for selecting households by portions determined for each tract. If the selected household was empty at the time or the selected resident did not agree to participate in the survey, the next house to the left was taken as a replacement.

At the household, a resident over 18 years of age was randomly selected to respond to the structured questionnaire applied through cell phones (iOS or Android) using the Epi Info^TM^ software, with daily exports, to compose the final database. Fieldwork was conducted by a coordinator in each municipality in partnership with the technician from the Regional Health Office covering the selected municipality.

In this study, information about the use of ivermectin to prevent COVID-19 was analyzed through the question: “did you take ivermectin to prevent COVID-19?”, with the following response options: “I already did that”, “I continue doing this”, “I intend to do it” and “I do not intend to do it”. For the analysis, the variable was dichotomized into no (“I do not intend to do it”) and yes (“I have already done it”, “I continue to do it” or “I intend to do it”). Only 49 individuals reported intending to use it, so they were allocated in the “yes” category.

The sociodemographic variables used were: health macro-region of the municipality (North, Central-North, East, West, South and Central-Northwest); size of municipality (small—up to 65,000 inhab.; medium—65,000 to 150,000 inhab.; and large—>150,000 inhab.); sex; age group (18 to 29, 30 to 49, 50 to 59, 60 and over); ethnicity/skin color (white, brown, black, yellow and indigenous); schooling (up to complete elementary school, incomplete and complete high school, complete higher education or more); and family income (less than one minimum wage (less than R$ 1,045.00), from one to less than three minimum wages (from R$ 1,045.00 R$ 3,134.99); three or more minimum wages (R$ 3,135.00 or more). Respondents were also asked about their current work situation (work, study, work and study, retired, housewife, does not work/does not study) and if any resident of the household received government financial benefit (yes/no). The change in monthly family income was evaluated based on measures of social distance, through the following categories: decreased completely or more than half, decreased by half, decreased by less than half, and did not decrease or increased.

It was also asked if the interviewee had already been infected (laboratory analysis) and diagnosed with COVID-19 and about presence of symptoms prior to the interview or at the moment. In addition, self-assessment of knowledge about COVID-19 was examined. The laboratory analysis was carried out using a commercial kit imported by Diasorin (Registration of the Ministry of Health: 103.398.40-56), from the Italian company Liaison^®^, under batch 354020 and expiry December 15, 2020; tests were performed using chemiluminescence, for the quantitative determination of antibodies of the immunoglobulin G (IgG) type against the S1 and S2 proteins of SARS-CoV-2, with a report by the supplier of 97.4% sensitivity (percentage of accuracy of positives) and 98.5% specificity (percentage of correct negatives). The authors also carried out an internal validation, in addition to complying with the biosafety protocols of the Central Public Health Laboratory of Mato Grosso (LACEN-MT) at all stages of testing. The choice of this test was made after accessing available commercial kits and internal quality evaluations.

As for knowledge about COVID-19 and its treatment, respondents were asked how they evaluated their knowledge about the disease as good/very good; regular; and poor/very poor. For the question “about the treatment of the disease, do you believe that…”, response options were: “there is a drug to treat the new coronavirus”; “there is a vaccine for the new coronavirus”; “there is medicine and vaccine for the new coronavirus”; “there is no medicine or vaccine for the new coronavirus”; and “I do not know”.

All analyses were performed using the Stata 12 software using the “svy” command, which allows the incorporation of weighting factors and considers the complex design of the sample. The sampling weight of each selected unit (census tract, household, and individual) was calculated separately for each municipality, considering the inverse of the probability of selection according to the study's sampling plan, and corrections for adjustments of known population totals were included. Variables were described using relative frequencies. Pearson's χ^2^ test was applied to assess the association between variables. A statistical significance level of 5% (α≤0.05) was considered.

All ethical aspects in research were respected, in accordance with Resolution 466/2012 of the National Health Council (CNS). This project is part of the matrix project “Endemic and Epidemic Diseases of Mato Grosso”, approved by the Research Ethics Committee (CEP) of the Universidade do Estado de Mato Grosso (UNEMAT), opinion 3.986.293/2020. All participants signed the Free and Informed Consent Form and were assisted in their homes following strict biosafety protocols.

## RESULTS

Of 4,306 visits (95% of the estimated sample), 4,206 were analyzed (92.8% of the estimated sample), and had blood samples evaluated. The prevalence of ivermectin use to prevent COVID-19 in Mato Grosso was 58.3%, being higher in the West region (66.6%) and lower in the North region (39.7%) of the state (Table 1).

**Table 1:** Prevalence of ivermectin use to prevent COVID-19, according to sociodemographic and economic variables. Mato Grosso, September/October 2020.

Variables		Overall		Use of ivermectin		p-value
				%*	95%CI†	
		n	%†	58.9	55.8; 61.93	
Health macro-region						
	West	105	2.5	66.6	60.2; 72.3	**0.012**
	North center	888	21.1	60.2	55.6; 64.6	
	East	1.686	40.1	60.1	53.6; 66.2	
	Northwest center	833	19.8	58.6	52.6; 64.4	
	South	366	8.7	55.5	47.5; 63.1	
	North	328	7.8	39.7	52.6; 64.4	
Municipality size						
	Small	1.997	47.5	58.9	53.2; 64.4	0.74
	Average	958	22.8	56.4	51.3; 61.3	
	Big	1.253	29.8	58.8	54.9; 62.7	
Sex						
	Female	1.943	46.2	62.9	57.9; 67.7	0.08
	Male	2.263	53.8	55.5	50.0; 60.9	
Age range (years)						
	18–29	736	17.5	50.1	39.1; 60.2	**0.04**
	30–49	1.787	42.5	59.0	52.9; 67.9	
	50–59	744	17.7	**69.7**	**63.5; 75.3**	
	60 and over	942	22.4	57.2	52.1; 62.1	
Skin color‡						
	White	1.404	33.48	**66.5**	**61.1; 71.5**	**<0.01**
	Brown	2.321	55.27	55.1	51.0; 59.1	
	Black	445	10.68	54.2	43.1; 64.8	
Education						
	Up to complete elementary school	1.728	41.1	51.7	46.1; 57.4	**<0.01**
	Incomplete and complete high school	1.766	42.0	61.7	56.6; 66.6	
	Complete higher education or more	706	16.8	**68.8**	**60.9; 75.7**	
Family income						
	Less than 1 minimum wage (less than R$ 1,045.00)	155	3.7	44.4	32.1; 57.4	**<0.01**
	From 1 to less than 3 minimum wages (from R$ 1,045.00 to R$ 3,134.99)	2.317	55.1	54.0	49.1; 58.9	
	3 or more minimum wages (R$ 3,135.00 or more)	1.732	41.2	**64.2**	**60.6; 68.4**	
Aid or benefits						
	Yes	2.813	66.9	64.1	58.0; 69.7	0.05
	No	1.392	33.1	56.4	52.6; 60.2	
Change in monthly household income with social distancing measures						
	Decreased entirely or in more than half	622	14.8	56.4	40.7; 70.9	0.51
	Decreased by half	564	13.4	55.9	45.2; 66.2	
	Decreased by less than half	522	12.4	67.2	58.6; 74.9	
	Did not decrease or increase	2.498	59.4	59.9	55.6; 64.1	

* Weighted percentage;

† 95% confidence interval;

‡ These two alternatives were excluded due to small number: yellow = 31 and indigenous = 3.

Bolded: variables with p-value <0.05 and categories with the highest prevalence.

The population was composed of adults (≥18 years), mostly men (53.8%), aged between 30 and 49 years (42.5%), 60 years and over (22.4%), and 50 to 59 years old (17.70%). The largest proportion of participants declared being brown (55.27%), with incomplete high school (42.0%), family income of one to three minimum wages (55.1%), and claimed to receive Government assistance or benefits (66.9%). When asked if there was a change in monthly family income, 59.4% said there had been no decrease or increase with the social distancing measures (Table 1).

There was no significant difference in the use of ivermectin between sexes and when it came to municipality size, income, and change in income, and in routine in the last month. On the other hand, the prevalence of drug use was higher among respondents aged 50 to 59 years, who declared being white, with higher education (higher education or more), and higher family income (three or more minimum wages). (Table 1).

Table 2 shows that 12.5% of the sample tested positive in the serological test for SARS-CoV-2 and 75.3% had no symptoms of the disease. When asked if they had already had COVID-19, 4.5% of respondents said they had been confirmed by the test. Only 24.8% of participants considered their knowledge of the new coronavirus to be poor or very poor. The prevalence of ivermectin use was higher among those who had a positive serological test for antibodies against SARs-CoV-2, those who reported previous symptoms of the disease, and those who considered their knowledge about COVID-19 to be good or very good. Of note was the fact that almost all cases who reported having been diagnosed with COVID-19 used ivermectin (91.2%).

**Table 2:** Prevalence of ivermectin use to prevent COVID-19, according to serological test results, previous diagnosis of COVID-19 and reported symptoms. Mato Grosso, September/October 2020.

Variables		Overall		Use of ivermectin		p-value
		n	%*	%*	95%CI†	
Serological test						
	Positive	522	12.4	**70.4**	**62.7; 77.0**	**<0.01**
	Negative	3.684	87.5	57.3	53.9; 60.6	
Presence of symptoms (n=3.958)						
	Yes	978	24.7	**72.4**	**66.0; 78.0**	**<0.01**
	No	2.980	75.3	54.4	50.2; 58.5	
Had COVID-19 previously (n=3.522)						
	No	2.546	72.3	56.2	51.6; 60.7	**<0.01**
	Not sure	694	19.7	57.7	50.7; 64.4	
	Yes, confirmed with a test	158	4.5	**91.2**	**83.4; 95.4**	
	I think so, I searched for a health service, but the test result was not ready	25	0.7	93.2	67.7; 98.9	
	I think so, I searched for a health service, but I didn't take the test	39	1.1	78.6	52.6; 92.4	
	I think so, I had the symptoms, but I didn't go to a health service	60	1.7	75.6	50.8; 90.3	
How the respondent rates their knowledge about the new coronavirus (prevention and characteristics) (n=3,727)						
	Very poor/poor	925	24.8	51.3	46.3; 56.2	**0.01**
	Adequate	1.383	37.1	57.2	52.3; 61.7	
	Very good/good	1.417	38.0	**65.0**	**57.7; 71.6**	
Knowledge about disease treatment						
	There is a drug to treat the new coronavirus	1.197	32.1	**66.9**	**60.1; 73.1**	**0.03**
	There is a vaccine for the new coronavirus	198	5.3	57.8	48.9; 66.0	
	There is a medicine and vaccine for the new coronavirus	496	13.3	60.3	46.7; 72.5	
	There is no medicine or vaccine for the new coronavirus	1.134	30.4	51.7	46.4; 58.9	
	I don't know	697	18.7	54.4	46.8; 61.9	

* Weighted percentage;

† 95% confidence interval.

Bolded: variables with p-value <0.05 and the most prevalent categories.

The prevalence of ivermectin use was higher among individuals who considered their knowledge about the disease to be good or very good (65.0%). Only 18% of respondents reported not knowing whether there was a treatment or vaccine for COVID-19, with a higher prevalence of ivermectin use among those who reported believing that there was already a drug approved to treat COVID-19 (66.9%).

## DISCUSSION

Through a home-based seroepidemiological survey carried out in Mato Grosso, it was estimated that 58.3% of respondents used ivermectin to prevent COVID-19, with differences between health regions of the state and greater use among older age groups, people with higher schooling, and income. It should be noted that most individuals who had COVID-19 antibodies, who reported having symptoms, and who had laboratory confirmation of the disease used the drug not proven effective until then.

The scientific literature has already shown a great demand for ivermectin to prevent and treat COVID-19^12,13^. The scenario of fear and anxiety imposed by the new coronavirus pandemic boosted a search for therapeutic and/or prophylactic pharmacological strategies, but the lack of a drug with proven efficacy against SARS-CoV-2 during the first year of the pandemic encouraged the population to use drugs still under evaluation for the virus^14^. However, it was found that abdominal pain, diarrhea, and taste alteration were more frequent among COVID-19 cases who received multiple doses of this drug^15^, and abdominal pain among those who received a single dose^16^. Furthermore, high doses of ivermectin have been associated with hypotension and neurological effects such as decreased consciousness, confusion, hallucinations, seizures, coma, and death^17^. There are still potential risks in the use of ivermectin, including skin, systemic, and ophthalmological reactions^18^.

In Brazil, few studies have evaluated self-medication in the context of COVID-19 and included ivermectin among the evaluated drugs^3,7^. In the present study, it was not possible to differentiate the use of ivermectin as self-medication or after medical prescription, and more than half of the interviewees used the medication. This result was higher than those estimated in a survey carried out in Manaus, in which 38.6% of individuals self-medicated to prevent SARS-CoV-2 infection, mostly with azithromycin and ivermectin^9^.

This study identified that the use of ivermectin was higher among individuals aged 50 to 59 years (69.7%), which may be related to the fact that this age group is considered economically active and has a higher risk for disease aggravation, and is feeling afraid of getting the most severe form of the disease, so they end up taking medications with the intention of preventing or treating COVID-19^19^.

Still, regarding the profile of ivermectin users in the studied context, the highest prevalence was among those who declared themselves to be white, with higher education, and earning three or more minimum wages. These factors may indicate greater distrust of official information or lower perception of disease risk among individuals of higher socioeconomic status, similar to what has already been discussed in previous studies regarding possible explanations for refusing vaccination, which include, in addition to these factors, multiple and divergent sources of information available^19,20^. This hypothesis is reinforced by the result of greater use of the medication among individuals who considered their knowledge about the disease to be good or very good—since the information is not always reliable, given the polarization of the discussion on COVID-19; the large-scale, deliberate and intentional production of fake news about the pandemic deserves attention, as the aim was to deceive, manipulate, and deny reality for political and economical reasons^21^.

In Mato Grosso, the “COVID kit” was also adopted by the state government, which acquired a large number of batches of medicines to distribute among municipalities. The justification given by the government was to allow the “early treatment” of patients and prevent them from arriving in a serious condition at health facilities^7^.

Early treatment with drugs from the so-called “COVID kit” has always been dubious and had low methodological quality in clinical studies, producing unreliable estimates of efficacy and safety, there being no evidence of its effectiveness at the end of the first year of the pandemic^22^. The possibility that the efforts and resources employed by the public power in its promotion have been directed to reducing adherence to vaccination and non-pharmacological protection measures in the country is discussed^23,24^.

Some of the limitations of the present study are the fact that there is no differentiation between self-medication and medical advice, the failure to evaluate the dose and duration of use and, mainly, the chronology of use in the presence of symptoms or diagnosis, that is, it was not possible to differentiate preventive use from therapeutic use of ivermectin. Although the question posed to the interviewee relates only to its use to prevent COVID-19, the possibility that individuals have positively reported the use after the appearance of COVID-19 symptoms with a confirmed diagnosis of the disease is not ruled out.

Based on the researched literature, this is the first home-based study in the Midwest Region that evaluated the use of medicines for the prevention of COVID-19 by the population, which is a strength. A sample calculation that allows inferences for the population of ten municipalities in Mato Grosso was adopted, as these are polarizing centers due to the urban structure and the intensity of flows in existing networks, representing 47.37% of the total population of the state^8^.

We conclude that this characterization of high prevalence of ivermectin use by the population of the ten municipalities surveyed in the state of Mato Grosso leads to a profile with a higher age group, white skin color, high educational level, and income. This result may be related to the dissemination of fake news about the effectiveness of the drug for the prevention of COVID-19 addressing the so-called “early treatment” instead of preventive measures, a symbol of political bias in facing the pandemic.

This study is believed to be able to help health authorities to continue to intensify and promote effective measures to control the disease according to the epidemiological moment, using vaccination, social distancing, masks, and sanitary hygiene protocols, as well as to implement strategies to inform the population about the risks of off-label use of drugs and to fight the advertising of drugs indicated for the prevention and treatment of COVID-19 without proper proof of safety and efficacy.
